# The ciliary neurotrophic factor induces Stat3 phosphorylation in distinctive cytotypes of organs involved in body metabolism: An immunohistochemical study

**DOI:** 10.1111/joa.70195

**Published:** 2026-06-25

**Authors:** Chiara Galli, Georgia Colleluori, Jessica Perugini, Edoardo Scopini, Ilenia Severi, Gaia Grandin, Antonio Giordano

**Affiliations:** ^1^ Department of Experimental and Clinical Medicine Marche Polytechnic University Ancona Italy; ^2^ Center of Obesity Marche Polytechnic University Ancona Italy; ^3^ IRCCS INRCA Ancona Italy

**Keywords:** adipose tissue, CNTF receptor, gut, JAK–STAT, liver, pancreas, skeletal muscle, stomach

## Abstract

Administration of ciliary neurotrophic factor (CNTF) reduces food intake and body weight in both humans and experimental animals, where it also ameliorates hyperglycemia, hyperinsulinemia, and dyslipidemia. To exert its anti‐obesogenic and anti‐diabetogenic effects, CNTF targets brain feeding centers as well as multiple peripheral organs inducing the phosphorylation of the transcription factor signal transducer and activator of transcription 3 (p‐STAT3). However, data showing which peripheral cytotypes are specifically targeted by exogenous CNTF in vivo in metabolically relevant organs are currently lacking. Here, we first evaluated the gene expression levels of the subunits of the tripartite CNTF receptor (Cntfr) complex, that is, the *Cntfrα*, the leukemia inhibitory factor receptor β (*Lifrβ*) and the glycoprotein 130 (*gp130*), by quantitative real‐time PCR in metabolically relevant organs of adult male mice: gastrointestinal (GI) tract, pancreas, liver, visceral and subcutaneous white (WAT) and interscapular brown adipose tissue (iBAT), skeletal muscle and the sciatic nerve. We then quantified p‐STAT3 by Western blotting in these organs after intraperitoneal administration of CNTF (0.3 mg/kg) or saline. Finally, we mapped CNTF‐responsive cells by immunohistochemistry, followed by morphometric quantification and confocal microscopy in both CNTF‐ and saline‐treated mice. *Lifrβ* and *gp130* were ubiquitously detected across all the investigated organs; the *Cntfrα* showed the highest expression levels in the skeletal muscle, sciatic nerve, and iBAT, whereas it was found to be expressed to a lesser extent in the other sites. Administration of CNTF led to a significant increase of p‐STAT3/STAT3 protein ratio in all organs examined, except the duodenum, and induced a distinctive pattern of cell nuclear p‐STAT3 immunoreactivity. Notably, along the analyzed GI tract, CNTF induced nuclear STAT3 phosphorylation in neurons of the submucosal and myenteric plexuses of the enteric nervous system and in contractile cells of the muscularis externa, where the response peaked in the mesenteric gut and colon. In the pancreas, CNTF triggered a higher activation within the endocrine component compared to the exocrine parenchyma. In the liver, CNTF induced STAT3 phosphorylation not only in parenchymal cells but also in sinusoids and resident macrophages. The cytokine activated p‐STAT3 in subcutaneous and visceral white adipocytes, but also in brown adipocytes, with a prominent response observed in the beige subcutaneous adipocytes; adipose‐resident macrophages and endothelial cells of numerous blood vessels were also CNTF‐responsive. Lastly, in skeletal muscle, a major site for glucose/lipid utilization, CNTF induced widespread nuclear p‐STAT3 immunoreactivity in muscle fibers and in connective and Schwann cells of the peripheral nerves, including the sciatic nerve, supplying the gastrocnemius. In conclusion, our data indicate that CNTF acts across diverse cytotypes within metabolically relevant organs and tissues, likely fostering its peripheral metabolic effects through this cellular heterogeneity.

## Graphical abstract

p‐STAT3‐positive cells in metabolically relevant peripheral organs and tissues from mice administered with recombinant CNTF (0.3 mg/kg).

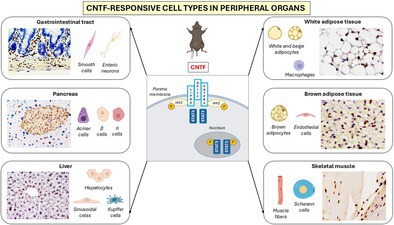

## 1 INTRODUCTION

The ciliary neurotrophic factor (CNTF) belongs to the interleukin (IL)‐6 cytokine family, a group of structurally related cytokines including IL‐6, IL‐11, leukemia inhibitory factor (LIF), oncostatin M, cardiotrophin 1 (CT‐1), cardiotrophin‐like cytokine (CLC), and IL‐27 (Rose‐John, 2018). By autocrine, paracrine, and/or endocrine signaling, IL‐6 cytokine family members affect several pathophysiological processes in mammals, ranging from the modulation of the immune response, especially B‐cell stimulation and induction of the hepatic acute‐phase proteins, to neurotrophic effects and coordination of body metabolism (Miyaoka et al., 2006, Ghanemi & ST‐Amand, 2018, Murakami et al., 2019).

CNTF was originally discovered as a growth factor supporting the survival of chick ciliary ganglion neurons (Adler et al., 1979). Later, it was demonstrated to display significant neurotrophic effects for a wide variety of central and peripheral neurons, including motor neurons (Sendtner et al., 1994; Sleeman et al., 2000). To exploit possible therapeutic effects of CNTF, patients affected by amyotrophic lateral sclerosis were chronically treated with a recombinant human CNTF (Miller et al., 1996). These patients did not show amelioration of motor performance; however, they experienced anorexia and weight loss among other side effects. Subsequent clinical and pre‐clinical studies confirmed that CNTF administration results in decreased food intake and weight loss and an improvement of obesity‐associated hyperglycemia, hyperinsulinemia, and dyslipidemia in humans and in animal models (Bluher et al., 2004; Lambert et al., 2001; Sleeman et al., 2003). On a mechanistic level, administered CNTF reduces food intake by acting on hypothalamic and brainstem feeding centers (Anderson et al., 2003; Lambert et al., 2001; Senzacqua et al., 2016; Venema et al., 2020), but also improves obesity‐associated metabolic alterations by acting on several metabolically relevant organs including liver, pancreas, skeletal muscle, and adipose tissue (Pasquin et al., 2015). However, a detailed anatomical description aimed at detecting which cytotypes are specifically targeted by exogenous CNTF in metabolically relevant organs is lacking.

The IL‐6 family cytokines bind to multimeric receptor complexes (Rose‐John, 2018). For CNTF, the cytokine binds to a three‐part receptor complex consisting of the ligand‐specific binding subunit receptor α (Cntfrα), the signal‐transducing subunit (gp130), and the LIF receptor β (Lifrβ) (Davis et al., 1993; Ip et al., 1993). While gp130 is almost ubiquitously expressed by mammalian cells and the LIF receptor is common also to LIF, CT‐1 and CLC signaling, the Cntfrα confers specificity to CNTF signaling (Sleeman et al., 2000). Importantly, CNTF activation of its tripartite receptor involves the activation of the janus family of tyrosine kinases (JAK1/JAK2) and signal transducers and activators of transcription (STAT), mainly STAT3 (Heinrich et al., 1998; Simi & Ibanez, 2010). STAT proteins are DNA‐binding factors activated by JAK proteins through tyrosine phosphorylation and dimerization in response to extracellular signals, in turn causing the translocation of STAT dimers from the cytoplasm to the nucleus (Aaronson & Horvath, 2002). While systemic CNTF has a very short half‐life (of the order of a few minutes as reported by Dittrich et al. (1994)), the rapid nuclear translocation of p‐STAT3, typically detected 45 min post‐injection, provides a highly sensitive frame of the direct cellular response even after systemic clearance has occurred (Anderson et al., 2003). Importantly, several studies previously demonstrated that STAT3 phosphorylation is induced by exogenous CNTF administration in both the central nervous system (CNS) (Severi et al., 2015; Venema et al., 2020) and in peripheral organs (Perugini et al., 2019; Rezende et al., 2009; Zvonic et al., 2003), and that this signaling pathway is the main responsible of CNTF effect (Rezende et al., 2009). The detection of the nuclear p‐STAT3 immunoreactivity after a single CNTF injection is hence a reliable anatomical tool for the characterization of CNTF‐responsive cells, similarly to what performed for the study of other cytokines including the transforming growth factor‐β (Liu et al., 2013) and leptin (Frontini et al., 2008; Hubschle et al., 2001).

In the present study, CNTF was administered via a single intraperitoneal (i.p.) injection at a dose of 0.3 mg/kg, a paradigm widely established in the scientific literature (Lambert et al., 2001) as it effectively mimics the anorectic and weight‐regulatory effects of the cytokine without inducing the acute‐phase inflammatory responses associated with higher dosages (Sleeman et al., 2003). With the primary aim of mapping cells responsive to exogenous CNTF at the anatomical level, we first quantified the expression of the tripartite receptor complex subunits *Cntfrα*, *Lifrβ* and *gp130* by quantitative real‐time PCR (qRT‐PCR) in murine peripheral organs playing crucial physiological functions in the regulation of the energy balance, such as nutrient digestion, absorption, metabolism, and storage (Yamada et al., 2008). We then quantified STAT3 activation by Western blotting and detected CNTF‐responsive cells, that is, cells bearing the functional CNTF receptor, by immunohistochemistry and morphometry in organs and tissues from CNTF‐treated and saline‐injected mice. Lastly, the phenotype of CNTF‐responsive cells was assessed by double labelling and confocal microscopy using well‐established cell type markers. Collectively, our data characterized the specific cellular targets of CNTF within peripheral metabolic organs, providing a structural basis to understand its physiological role in the regulation of energy balance.

## 2 MATERIALS AND METHODS

### 2.1 Animals and experimental conditions

Two‐month‐old male C57BL/6 mice were purchased from Charles River and housed individually under constant environmental conditions, with a 12‐h light/dark cycle at 22°C and ad libitum access to standard chow diet and water. Animals were deeply anesthetized with isoflurane and sacrificed in a fed state between 11:00 am and 12:00 pm. The principles of good laboratory animal care practice were followed, and experiments were conducted in accordance with the Council Directive 2010/63/EU of the European Parliament. All experiments were approved by the Italian Ministry of Health (authorization number: 96E38).

### 2.2 Tissue collection and processing

For qRT‐PCR analysis, anesthetized animals (*n* = 4) were decapitated. For Western blotting, mice received a single i.p. injection of either recombinant rat CNTF (0.3 mg/kg of body weight; R&D Systems, #557‐NT; *n* = 3) or an equal volume of saline (CTRL; *n* = 3). The injected volumes of CNTF or saline were kept equal according to body weight and were performed using a Hamilton syringe. Forty‐five minutes post‐injection, treated and control mice were sacrificed by decapitation. For both experimental settings the following samples were collected: stomach, duodenum, mesenteric intestine (jejunum and ileum), colon, liver, pancreas, inguinal white adipose tissue (iWAT), epididymal white adipose tissue (eWAT), interscapular brown adipose tissue (iBAT), the gastrocnemius skeletal muscle, and the sciatic nerve. Following the dissection, all specimens were snap‐frozen in liquid nitrogen and stored at −80°C until further use.

For morphological analyses, mice were treated with CNTF (*n* = 3) or saline (CTRL; *n* = 3) as described above. Forty‐five minutes post‐injection, animals were anesthetized and transcardially perfused with 4% paraformaldehyde in 0.1 M phosphate buffer (PB), pH 7.4 and the above listed organs were collected. After post‐fixation in 4% paraformaldehyde for 24 h at 4°C, specimens were dehydrated with increasing alcohol concentration, cleared with xylol, and embedded in paraffin.

### 2.3 qRT‐PCR

Total RNA was extracted with Trizol reagent (Invitrogen; #15596018), purified, digested with ribonuclease‐free deoxyribonuclease, and concentrated using the Total RNA purification kit (Norgen Biotek Corp.; #17250) according to the manufacturer's instructions. For determination of mRNA levels, 1 μg of RNA was reverse‐transcribed with the High‐Capacity cDNA RT Kit with RNase Inhibitor (Applied BioSystems; #4374967) in a total volume of 20 μL. qRT‐PCR was performed using TaqMan Gene Expression Assays and TaqMan Master Mix (Applied BioSystems; #4304437). All probes (Table S1) were purchased from Applied BioSystems. Reactions were carried out by the Step One Plus Real Time PCR system (Applied BioSystems) using 50 ng cDNA in a final reaction volume of 10 μL. The thermal cycle protocol consisted of initial incubation at 95°C for 10 min followed by 40 cycles of 95°C for 15 s and 60°C for 20 s. All samples were run in duplicate. Samples not containing the cDNA template were included as negative controls in all experiments. TATA box‐binding protein (Tbp) was selected as a housekeeping gene to normalize gene expression. Relative mRNA expression was determined by the Δ*C*_t_ method ($2^{-∆C_{t}}$).

### 2.4 Western blotting

Tissue lysates were prepared using a lysis buffer containing 50 mM Tris‐HCl (pH 7.4), 1% NP‐40, 1 mM EDTA, 150 mM NaCl, 1 mM sodium orthovanadate, 0.5% sodium deoxycholate, 0.1% SDS, 2 mM phenylmethylsulfonylfluoride, and 50 mg/mL aprotinin. Samples were centrifuged, and protein concentrations were determined by the Bradford Protein Assay (Bio‐Rad Laboratories, Segrate, Italy). Equal amounts of protein were separated by SDS‐PAGE and transferred onto nitrocellulose membranes using the Trans‐Blot TurboTM Transfer system (Bio‐Rad). To verify loading and transfer efficiency, membranes were visualized with Ponceau S solution (Santa Cruz Biotechnology, Santa Cruz, CA, USA). Membranes were then blocked for 1 h at room temperature (RT) in TBS‐T (50 mM Tris–HCL [pH 7.6], 200 mM NaCl, and 0.1% Tween‐20) containing 5% non‐fat dried milk, followed by overnight incubation at 4°C with the primary antibody (Table S2a). After washing in TBS‐T, membranes were incubated for 1 h at RT with the appropriate HRP‐conjugated secondary antibody (Table S2b). Immunoreactive bands were visualized using the Clarity™ Western ECL substrate and the Chemidoc Imaging System (all from Bio‐Rad). Densitometric analysis of bands was performed using Bio‐Rad Image Lab software. Where appropriate, membranes were stripped, washed, and re‐probed for total protein content.

### 2.5 Peroxidase immunohistochemistry

Peroxidase immunohistochemistry was performed on 4 μm thick paraffin‐embedded sections. To minimize procedural variability in the detection of p‐STAT3 immunoreactive cells, sections of organs from vehicle‐ and CNTF‐treated mice were exposed to immunoperoxidase procedures in parallel. Sections were rehydrated and then subjected to an unmasking procedure at 95°C for 20 min in an antigen retrieval solution (Bio‐Optica; #DV2004G1). Then, sections were treated with 3% H_2_O_2_ (in dH_2_O; 5 min) to block endogenous peroxidase, rinsed with phosphate‐buffered saline (PBS), and incubated in a 3% normal goat serum (Vector Laboratories; #S‐1000; in PBS, 20 min). Sections were then incubated overnight at 4°C with the monoclonal rabbit p‐STAT3 antibody (Table S2a), which served as our primary reference for all peroxidase‐based detections. After a thorough rinse in PBS, sections were incubated in the anti‐rabbit IgG biotinylated solution (Table S2b) in PBS for 30 min. Histochemical reactions were performed using Vectastain ABC kit (ABC Kit Elite Peroxidase Standard, Vector Laboratories; #PK6100) and 3,3′‐diaminobenzidine as substrate (DAB Substrate kit Peroxidase, Vector Laboratories; #SK4105). Sections were finally counterstained with hematoxylin, dehydrated, and mounted in Eukitt (Bio‐Optica). To assess the specificity of the antibody, negative controls were obtained by omitting the primary antibody. The immunohistochemical staining was analyzed using a Nikon Eclipse E600 microscope (Nikon).

### 2.6 Immunofluorescence and confocal microscopy

Immunofluorescence was performed on paraffin sections as follows: after dehydration, slices were washed in PBS containing 0.1% Tween for 5 min and then treated with a retrieval solution (Nacalai Tesque; #06380‐05; 1:10 in dH_2_O; pH 7) at 70°C for 40 min. Then, sections were treated with a blocking solution (Nacalai Tesque; #06349‐64) at RT for 40 min. Antibodies were applied overnight at 4°C. For immunofluorescence experiments, the choice between the monoclonal rabbit or the mouse p‐STAT3 antibody (Table S2a) was determined by the host species of the specific cellular markers used for double or triple staining, ensuring the absence of cross‐reactivity. Specifically, the mouse anti‐p‐STAT3 antibody was employed for colocalization studies in the mesenteric gut, liver, iWAT, and iBAT. The next day, sections were incubated in fluorophore‐linked secondary antibodies solution (Table S2b) in PBS for 1 h. Nuclear staining was performed using the fluorescent dye TO‐PRO‐3 Iodide (642 nm of excitation wavelength) (Invitrogen by Thermo Fisher; #T3602) in PBS for 15 min. Sections were subsequently mounted on standard glass slides and covered using Vectashield mounting medium (Vector Laboratories; #H‐1000‐10). Colocalization between antibodies was analyzed by a motorized Leica DM6000 microscope at 40X and 60X magnifications. Fluorescence was detected with a Leica TCS‐SL spectral confocal microscope equipped with an Argon and He/Ne mixed gas laser. Fluorophores were excited with the 488, 543 and 649 nm lasers and imaged separately. Images (1024 × 1024 pixels) were obtained sequentially from two channels using a confocal pinhole of 1.1200 and stored as TIFF files. The brightness and contrast of the final images were adjusted using Photoshop6 (Adobe Systems, RRID:SCR_014199).

### 2.7 Morphometric analysis

Morphometric analyses were performed on immunoperoxidase‐stained sections counterstained with hematoxylin. The percentage of p‐STAT3‐positive nuclei on the total number of nuclei was calculated in the specimens from CNTF‐treated (*n* = 3) and CTRL (*n* = 3) mice. For each organ, 3 to 5 non‐consecutive sections (at least 50 μm apart) were randomly selected and imaged using a Nikon Eclipse E600 microscope at 40X or 60X magnification. Within each section, at least five randomly selected different areas were analyzed, and a total of 500–1000 nuclei for each organ were manually counted using ImageJ. To ensure unbiased quantification, all acquired images were assigned a unique numerical code and randomized by an independent researcher prior to analysis. Therefore, the investigator performed p‐STAT3‐positive cell counting unaware of the specific experimental group assignments until the final data decoding. Nuclei were classified as p‐STAT3‐positive based on the presence of a distinct, dark brown reaction product.

### 2.8 Statistical analysis

Data are reported as mean ± standard error of the mean (SEM). Normality of data distribution was assessed using the Shapiro–Wilk test. For comparisons between two groups within a single tissue type, a two‐tailed Student's *t*‐test was used. For comparisons involving multiple groups or organs/compartments, a one‐way ANOVA followed by Tukey's post hoc test was employed. Statistical significance was set at *p* < 0.05. All analyses were performed using GraphPad Prism 6 software (RRID:SCR_002798).

## 3 RESULTS

### 3.1 Expression of *Cntfrα, Lifrβ*, and *gp130* transcripts in metabolically relevant peripheral organs

**FIGURE 1:**
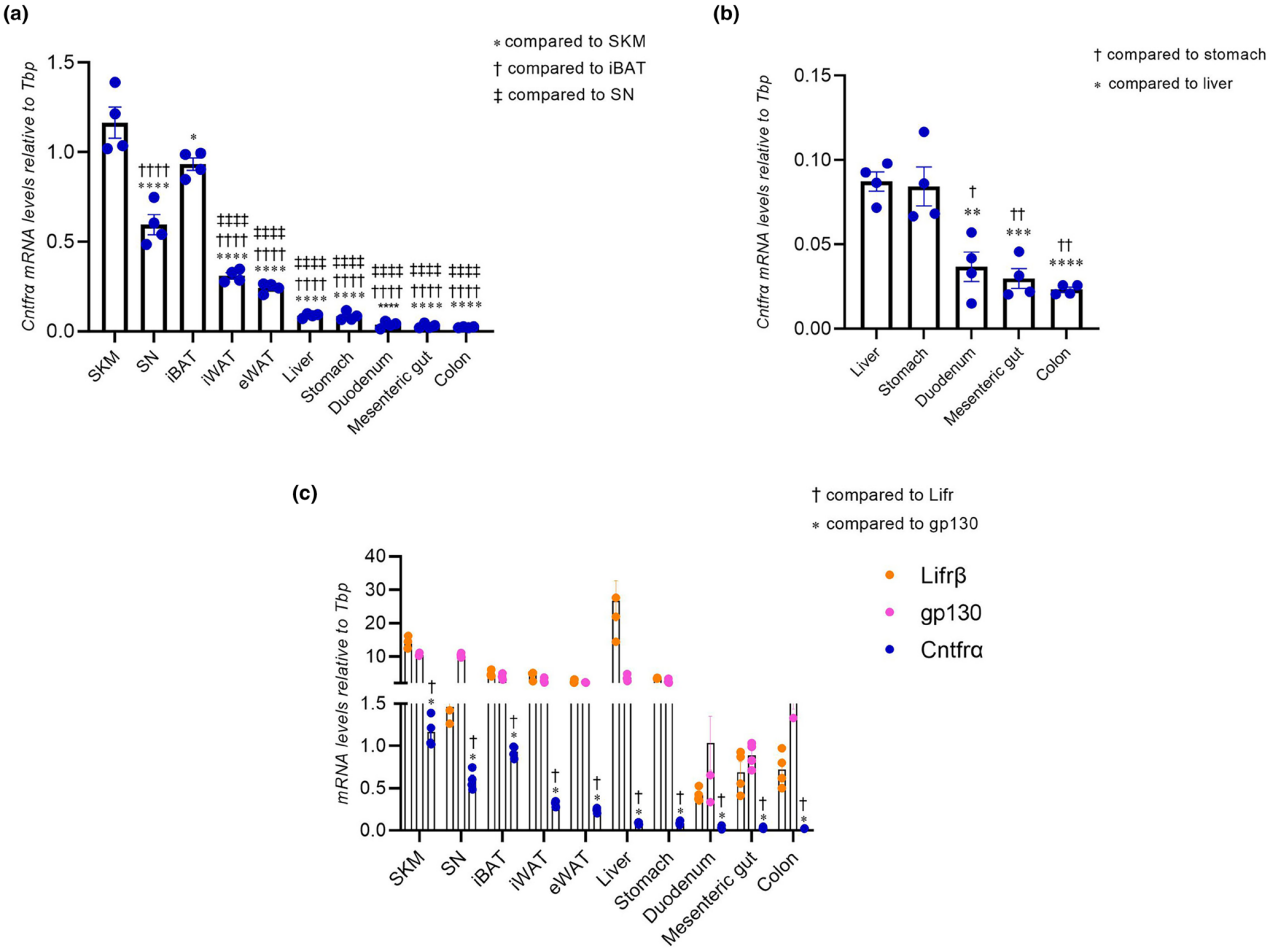
Cntfrα, Lifrβ and gp130 mRNA in murine metabolically relevant peripheral organs. (a) qRT‐PCR analysis of *Cntfrα* expression levels in skeletal muscle (SKM), sciatic nerve (SN), interscapular brown adipose tissue (iBAT), inguinal white adipose tissue (iWAT), epididymal white adipose tissue (eWAT), liver, stomach, duodenum, mesenteric gut and colon. Panel (b) shows a higher magnification of the organs with the lowest expression levels of *Cntfrα* to better appreciate the differences between the liver, stomach, and intestinal segments (duodenum, mesenteric gut and colon). (c) Comparative analysis of the mRNA expression of *Cntfrα* (blue) with *Lifrβ* (orange) and *gp130* (magenta). Data are presented as mean ± SEM (*n* = 4). In panels (a, b), the number of symbols indicates the level of significance: Single *p* < 0.05; double *p* < 0.01; triple *p* < 0.001; quadruple *p* < 0.0001. In panel (c), statistical significance is indicated by a single symbol representing *p* < 0.0001 due to graph scale constraints. Data were analyzed using two‐way ANOVA followed by Tukey's post hoc test. *Tbp* = TATA box‐binding protein.

Among the metabolically relevant peripheral organs analyzed, the skeletal muscle displayed the highest expression of *Cntfrα*, followed by a substantial expression in the sciatic nerve and iBAT (Figure 1a). Among the alimentary system organs, the liver and stomach showed the most prominent expression levels, whereas the duodenum, mesenteric gut, and colon displayed progressively lower transcript abundance (Figure 1b). The quantification of pancreatic *Cntfrα* was hampered by the high intrinsic ribonuclease activity typical of this tissue (Al‐Adsani et al., 2022; Augereau et al., 2016; Azevedo‐Pouly et al., 2014), which precluded the recovery of high‐quality mRNA. To circumvent this technical limitation, we performed a cross‐check with the publicly available scRNA‐seq dataset, *Tabula Muris* (Tabula Muris, 2018). This analysis confirmed a robust and selective expression of *Cntfrα* within the endocrine pancreatic islets, providing an independent validation of the receptor's presence in this organ. Notably, these available datasets, documenting *Cntfrα* expression in the liver, skeletal muscle, and adipose tissues, were also consistent with our results. In this dataset, however, the lack of *Cntfrα* detection in the other organs (i.e., stomach) may be attributed to the relatively low expression levels of *Cntfrα* in the gastrointestinal tract (GI), as well as to the well‐known lower sensitivity of scRNA‐seq compared to qRT‐PCR (Kolodziejczyk & Lonnberg, 2018). Collectively, these data highlight a pronounced different quantitative distribution of the *Cntfrα* transcript in metabolically relevant peripheral organs.

Given that CNTF functional signaling requires the assembly of Cntfrα with the two signal‐transducing subunits, gp130 and Lifrβ, we quantified their mRNA levels across all examined peripheral organs using qRT‐PCR. Both *gp130* and *Lifrβ* displayed a quantitatively notable and ubiquitous expression throughout the peripheral tissues (Figure 1c). This molecular profiling indicates that, while *Cntfrα* shows a more specific tissue distribution, the ubiquitous expression of *gp130* and *Lifrβ* identifies these organs as broad targets for the IL‐6 family cytokines.

### 3.2 Detection and characterization of CNTF‐responsive cells in metabolically relevant peripheral organs

To investigate the peripheral targets of exogenous CNTF, we mapped the cellular distribution of p‐STAT3 across organs essential for nutrient handling, energy storage, and expenditure. Specifically, we performed a systematic anatomical and morphometric analysis of the GI tract, liver, pancreas, adipose tissues, and skeletal muscle. The anatomical description, combined with the results from our morphometric analyses, is summarized in Table 1.

**TABLE 1:** Summary of p‐STAT3‐positive nuclei distribution and quantification across peripheral organs.

Organ	Anatomical site	Cytotype	Marker	% of total p‐STAT3(+) nuclei − CTRL	% of total p‐STAT3(+) nuclei − CNTF	Δ %	*p*
Stomach	Submucosa	Connective and endothelial cells	Morphology	6% ± 2%	19% ± 3%	13% ± 3%	0.0003
	Muscular layer	Smooth muscle cells	Morphology	6% ± 2%	29% ± 4%	23% ± 5%	< 0.0001
Duodenum	Submucosa	Brunner's glands	Morphology	6% ± 1%	22% ± 3%	16% ± 4%	0.0002
	Muscular layer	Smooth muscle cells	Morphology	2% ± 1%	38% ± 5%	36% ± 7%	0.0001
Mesenteric gut	Lamina propria	Connective, immune and endothelial cells	Morphology	1% ± 0.4%	27% ± 3%	26% ± 4%	< 0.0001
	Submucosa	Connective and endothelial cells	Morphology	5% ± 1%	34% ± 2%	30% ± 3%	< 0.0001
		Ganglia	PGP9.5				
	Muscular layer	Smooth muscle cells	Morphology	4% ± 1%	47% ± 3%	43% ± 4%	< 0.0001
		Ganglia	PGP9.5				
Colon	Lamina propria	Connective, immune and endothelial cells	Morphology	0.2% ± 0%	34% ± 6%	34% ± 6%	< 0.0001
	Submucosa	Connective and endothelial cells	Morphology	0.3% ± 0%	30% ± 3%	30% ± 3%	< 0.0001
		Ganglia	PGP9.5				
	Muscular layer	Smooth muscle cells	Morphology	1% ± 0%	53% ± 5%	52% ± 5%	< 0.0001
		Ganglia	PGP9.5				
Pancreas	Endocrine	α‐ and β‐cells	Insulin/Glucagon	2% ± 0%	43% ± 5%	41% ± 5%	< 0.0001
	Exocrine	Acinar cells	Morphology	13% ± 3%	26% ± 3%	13% ± 5%	0.01
Liver	Parenchyma	Hepatocytes	Morphology	3% ± 1%	53% ± 3%	50% ± 4%	0.0001
	Sinusoids	Endothelial cells	Morphology				
		Kupffer cells	F4/80				
WAT	Inguinal	White adipocytes	PLIN‐1	1% ± 0%	44% ± 2%	43% ± 2%	< 0.0001
		Macrophages	F4/80				
		Endothelial cells	PECAM‐1				
		Beige adipocytes	Morphology	0% ± 0%	59% ± 2%	59% ± 2%	< 0.0001
	Epididymal	White adipocytes	PLIN‐1	3% ± 1%	32% ± 2%	29% ± 3%	< 0.0001
		Macrophages	F4/80				
		Endothelial cells	PECAM‐1				
BAT	Interscapular	Brown adipocytes	UCP1	0.4% ± 0%	49% ± 2%	49% ± 12%	< 0.0001
		Endothelial cells	Morphology				
Skeletal muscle	Fibers	Myocytes	Morphology	3% ± 1%	36% ± 2%	33% ± 3%	< 0.0001
	Sciatic nerve	Schwann's cells	S100b	8% ± 1%	45% ± 3%	37% ± 4%	< 0.0001

Abbreviations: Δ = (difference between means), + = positive, CTRL = saline‐treated mice.

#### 3.2.1 Gastrointestinal tract

The CNTF administration triggered a widespread increase in STAT3 phosphorylation throughout the analyzed organs belonging to the GI tract.

**FIGURE 2:**
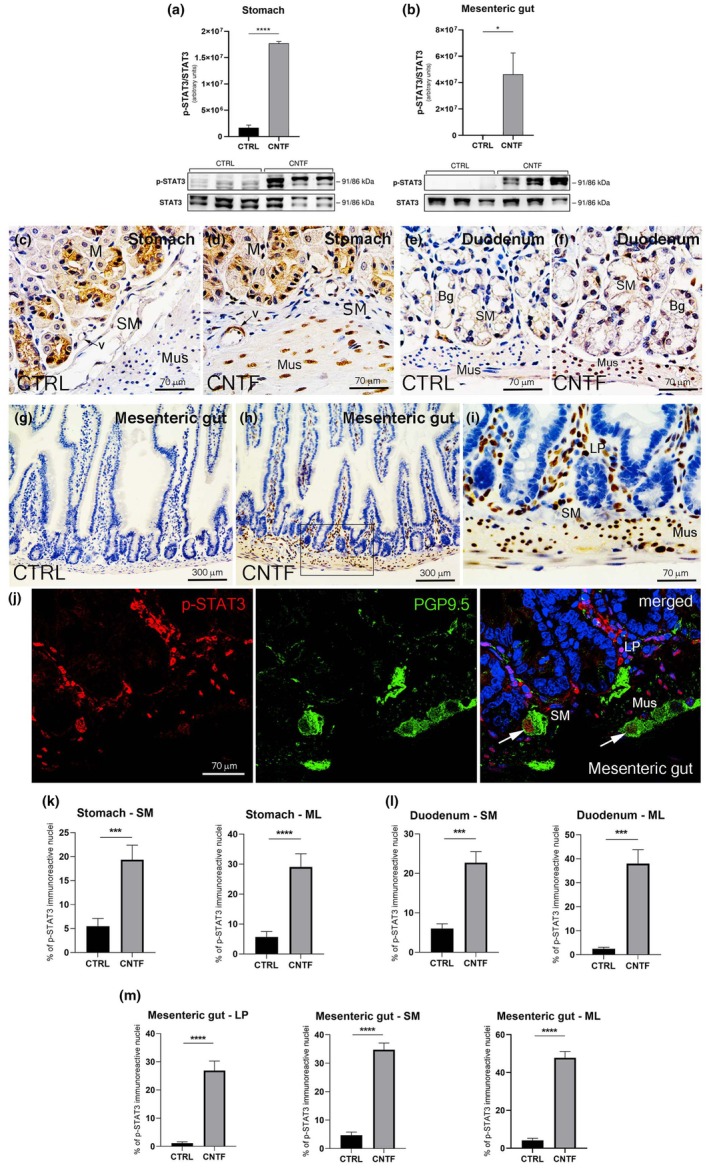
p‐STAT3 immunoreactivity after CNTF administration in murine gastrointestinal tract. (a) Western blot analysis and corresponding densitometric quantification of p‐STAT3 levels in the stomach and (b) mesenteric gut of saline‐ (CTRL) and CNTF‐treated mice. Total STAT3 was used as a loading control. Peroxidase immunohistochemistry for p‐STAT3 in the stomach (c, d), in the duodenum (e, f) and in the mesenteric gut (g, h) after saline or CNTF administration. Figure (i) is an enlargement of the corresponding areas framed in (h), showing a strong p‐STAT3 immunoreactivity in several nuclei of the lamina propria and of the muscularis externa. Double‐labelled confocal microscopy in the mesenteric gut of CNTF‐treated mice (j), showing the colocalization between p‐STAT3 and the pan‐neural marker PGP9.5 in ganglia of the enteric nervous system in both the submucosal and myenteric plexuses (white arrows). Morphometric quantification of the percentage of p‐STAT3‐immunoreactive nuclei in the different anatomical layers of the stomach (k), duodenum (l), and mesenteric gut (m). Data are expressed as mean ± SEM (*n* = 3 per group). *p < 0.05, ***p < 0.001, ****p < 0.0001 (Unpaired *t*‐test). Bg, Brunner's glands; LP, Lamina propria; M, Mucosa; Mus/ML, Muscularis layer; SM, Submucosa; v, Blood vessels.

In the stomach, Western blot analysis showed a very significant induction of p‐STAT3 protein levels (Figure 2a). Immunohistochemistry for p‐STAT3 revealed a basal cytoplasmic immunoreactivity restricted to the gastric mucosa in the stomach of saline‐treated mice (Figure 2c), as previously reported (Judd et al., 2006). However, this basal signal remained unaffected by CNTF treatment (Figure 2d). In contrast, CNTF specifically induced a significant p‐STAT3 nuclear translocation within the smooth muscle cells of the muscularis externa, submucosal connective cells, and vascular endothelial cells (Figure 2d). Quantitative morphometric analysis confirmed this activation, with p‐STAT3‐positive nuclei reaching 29% ± 4% (vs 6% ± 2% in the CTRL, *p* < 0.0001) in the muscular layer and 19% ± 3% (vs 6% ± 2% in the CTRL, *p* = 0.0003) in the submucosa (Figure 2k and Table 1).

In the duodenum, Western blot analysis failed to show a significant increase of p‐STAT3 protein levels; this lack of signal could not be attributed to protein degradation or poor sample quality, as the total STAT3 protein was clearly detectable and stable across all duodenal samples. In striking contrast, while immunohistochemistry in saline‐treated mice showed negligible p‐STAT3 immunoreactivity, except for a faint staining in some submucosal Brunner's gland cells (Figure 2e), CNTF administration induced a widespread nuclear p‐STAT3 immunoreactivity in cells of the muscularis externa (38% ± 5% vs. 2% ± 1% in the CTRL, *p* = 0.0001) and of the submucosal Brunner's glands (22% ± 3% vs. 6% ± 1% in the CTRL, *p* = 0.0002; Figure 2f, l and Table 1).

In the mesenteric gut, Western blotting revealed a significant increase of STAT3 phosphorylation following CNTF treatment (Figure 2b). Consistently, our immunohistochemical assessment showed that CNTF induced a strong STAT3 phosphorylation in cells of the lamina propria (27% ± 2% vs. 1% ± 0.4% in the CTRL, *p* < 0.001; Figure 2m and Table 1), the submucosa (34% ± 2% vs. 0.2% ± 0% in the CTRL, *p* < 0.001; Figure 2m and Table 1), and the muscularis externa (47% ± 3% vs. 4% ± 1% in the CTRL, *p* < 0.001; Figure 2m and Table 1). In these latter layers, p‐STAT3 was also observed in PGP9.5‐positive neurons of the submucosal and myenteric plexuses, respectively (Figure 2j).

Similar results were obtained in the colon, where Western blotting revealed a marked increase in p‐STAT3/STAT3 ratio upon CNTF treatment (Figure S1a), which activated lamina propria, submucosa, and myenteric ganglia cells (53% ± 5% vs. 1% ± 0% in the CTRL, *p* < 0.0001; Figure S1b–d and Table 1).

Both the small and the large intestine contain a population of mucosal enteroendocrine cells, which are the main source of incretins, such as the glucagon‐like peptide‐1 (GLP‐1) and gastric inhibitory peptide (GIP) (Hirasawa et al., 2005). GLP‐1 and GIP are produced by epithelial cells of the mucosa; in particular, GLP‐1 is secreted by L‐cells in both the small and the large intestine, while GIP is mainly secreted by K‐cells contained in the small intestine (Holst, 2004). However, we failed to observe p‐STAT3 immunoreactivity in GLP‐1‐ and GIP‐positive cells in these organs after CNTF administration by double staining immunofluorescence and confocal microscopy (Figure S2).

Overall, the present results indicate that administered CNTF does not act on epithelial cells lining the lumen of the analyzed GI tract segments. Instead, it targets specific cytotypes of the lamina propria, submucosa, muscularis externa, and the enteric nervous system, suggesting that CNTF influences gastrointestinal function indirectly by modulating, for example, Brunner's gland cell physiology and gut motility rather than affecting epithelium‐dependent secretory or absorbing functions.

#### 3.2.2 Pancreas

**FIGURE 3:**
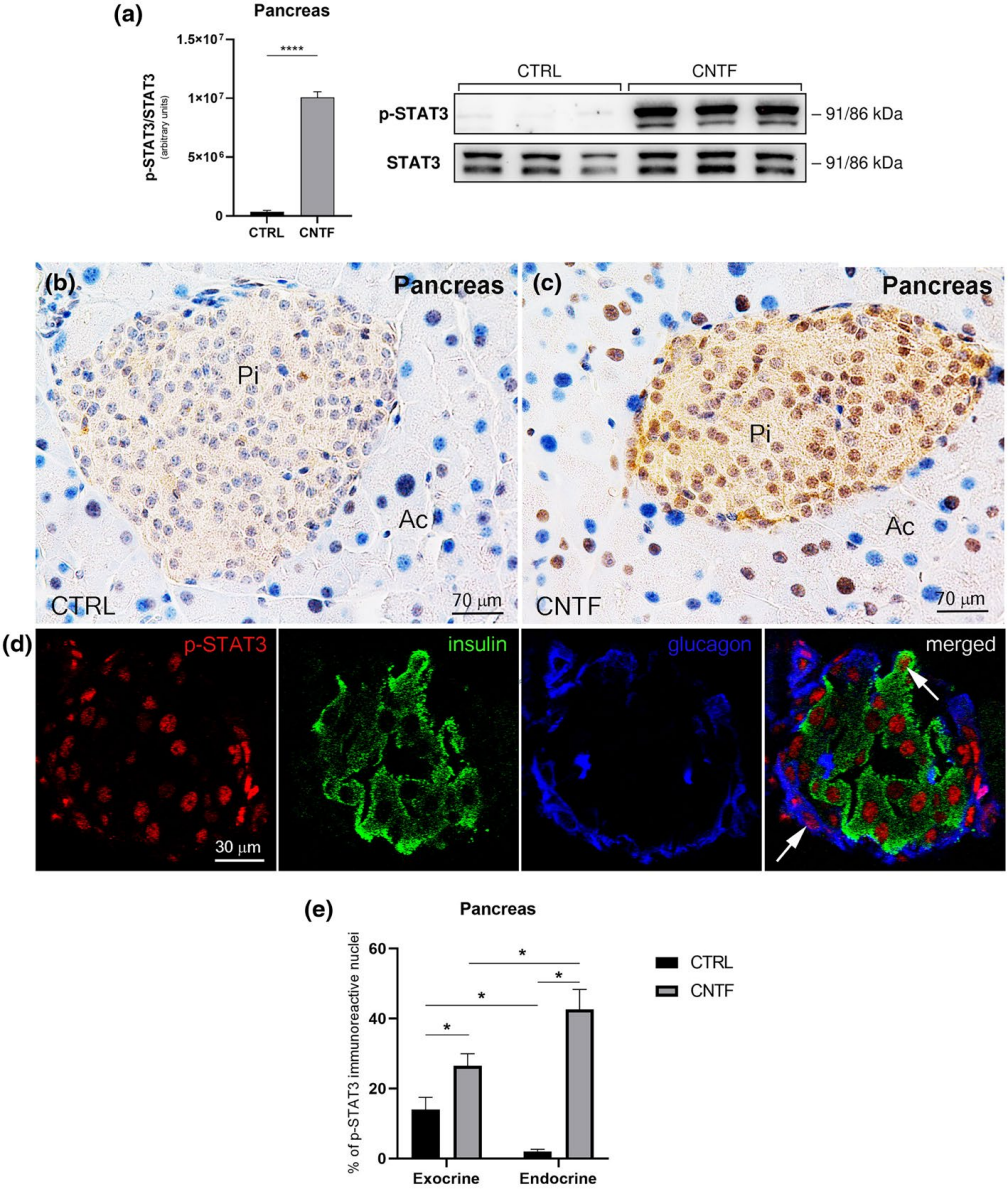
p‐STAT3 immunoreactivity after CNTF administration in murine pancreas. (a) Representative Western blot and relative densitometric quantification of p‐STAT3 protein levels in the pancreas of saline‐ (CTRL) and CNTF‐treated mice. Total STAT3 was used as a loading control. (b, c) Peroxidase immunohistochemistry for p‐STAT3 in pancreatic islets after saline or CNTF administration. (d) Double‐labelled confocal microscopy for p‐STAT3, insulin and glucagon (white arrows) in pancreatic islets after CNTF administration. (e) Morphometric quantification of p‐STAT3‐positive nuclei in the endocrine and exocrine components of the pancreas. Data are mean ± SEM (*n* = 3 per group). *p < 0.05; ****p < 0.0001; Unpaired *t*‐test for (a); Two‐way ANOVA followed by Tukey's post hoc test for (e). Ac = acinar cells, Pi = pancreatic islets.

To evaluate the activation of the JAK/STAT3 pathway in the pancreas, we first performed Western blot analysis on total protein extracts. Systemic CNTF administration induced a significant increase in p‐STAT3 protein levels compared to saline‐treated mice, where the signal was markedly low (Figure 3a). Immunohistochemistry in saline‐treated mice revealed the presence of several p‐STAT3‐positive cells scattered in the exocrine compartment, while the endocrine islets showed a nearly absent signal (Figure 3b). Interestingly, morphometric analysis in saline‐treated mice revealed a differential basal phosphorylation of STAT3 within the organ: the exocrine compartment displayed a significantly higher basal phosphorylation compared to the endocrine islets (13% ± 3% vs. 2% ± 0%, respectively, *p* < 0.05; Figure 3e and Table 1). On the other hand, the magnitude of the response to systemic CNTF administration was markedly more evident in the pancreatic islets (compare Figure 3c with 3b). In the endocrine compartment, CNTF induced a pronounced activation (reaching 43% ± 5% vs. 2% ± 0% in the CTRL, *p* < 0.0001; Figure 3e and Table 1), whereas the response in the exocrine pancreas was comparatively lower, but still significant (26% ± 3% vs. 13% ± 3% in the CTRL, *p* < 0.05; Figure 3e and Table 1). Murine pancreatic islets are composed primarily of insulin‐secreting β‐cells that in mice are clustered in a central core, surrounded by α‐cells secreting glucagon, which is the second most represented cytotype in the pancreatic islets (Steiner et al., 2010). As shown by triple staining and confocal microscopy, CNTF induced p‐STAT3 immunoreactivity in both insulin‐positive β‐cells and in glucagon‐positive α‐cells (Figure 3d). In summary, our data indicate that endocrine islets represent the most responsive CNTF‐target within the pancreas, while exocrine parenchymal cells display a weaker response.

#### 3.2.3 Liver

**FIGURE 4:**
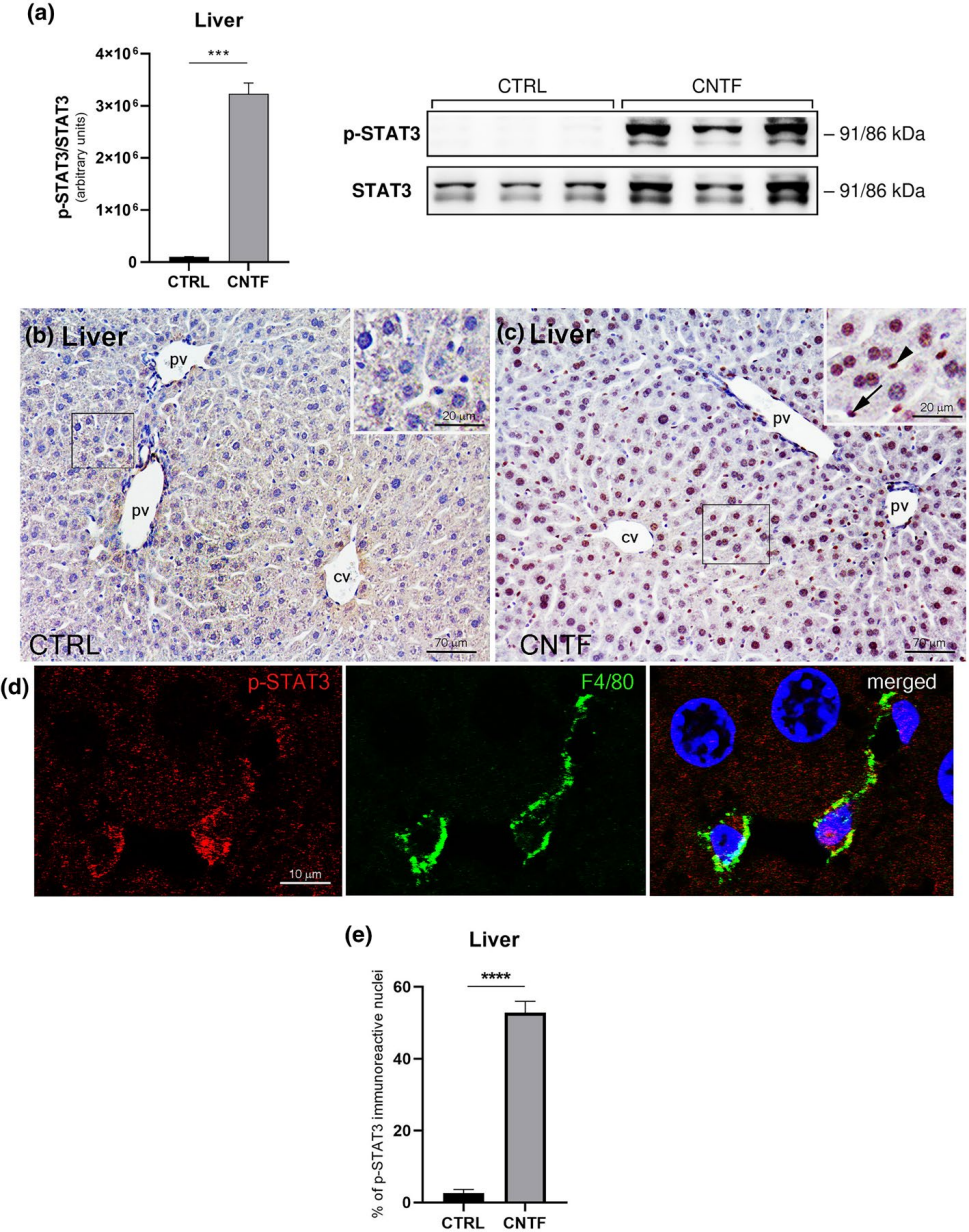
p‐STAT3 immunoreactivity after CNTF administration in murine liver. (a) Representative Western blot and relative densitometric quantification of p‐STAT3 protein levels in the liver of saline‐ (CTRL) and CNTF‐treated mice. Total STAT3 was used as a loading control. (b, c) Peroxidase immunohistochemistry for p‐STAT3 in the liver after saline or CNTF administration. Insets are enlargements of the corresponding framed areas in (b) and in (c). Figure (c) shows p‐STAT3 immunoreactivity induced by CNTF in polyploid and binucleated hepatocytes, in elongated cells at the wall of the hepatic sinusoids (arrowhead), compatible with endothelial cells, and in group of cells located inside the hepatic sinusoids (black arrow), resembling Kupffer's cells. (d) Double‐labelled confocal microscopy for p‐STAT3 and the macrophage marker F4/80 in the liver after CNTF administration. (e) Morphometric quantification of p‐STAT3‐positive nuclei in the liver. Data are mean ± SEM (*n* = 3 per group). ***p < 0.001; ****p < 0.0001; Unpaired *t*‐test; cv = central vein, pv = portal vein.

By Western blotting, in saline‐treated mice p‐STAT3 protein levels were virtually absent; CNTF administration induced a significant increase in STAT3 phosphorylation (Figure 4a). Consistently, immunohistochemical analysis in control mice revealed only a weak cytoplasmic p‐STAT3 immunoreactivity in the parenchyma of the liver, possibly due to non‐specific background (Figure 4b), while CNTF administration resulted in a strong STAT3 phosphorylation throughout the liver parenchyma (Figure 4c). Such observations were confirmed by morphometric analysis revealing only very few p‐STAT3 immunoreactive cell nuclei in saline‐treated mice (Figure 4e and Table 1), in stark contrast with a massive and widespread response in CNTF‐treated mice (53% ± 3% vs. 3% ± 1% in the CTRL, *p* < 0.0001; Figure 4e and Table 1). The p‐STAT3 immunoreactivity was predominantly localized in central and rounded nuclei of numerous polyhedral hepatocytes, forming the typical plates spaced by sinusoids (Figure 4c). Binucleated hepatocytes and hepatocytes containing large, likely polyploid nuclei were also p‐STAT3‐positive (Figure 4c, inset). In CNTF‐treated mice, p‐STAT3 immunoreactivity was also detected in other cell types than hepatocytes, including cells lining the wall of the sinusoids (endothelial cells) and other cells attached to the wall of the sinusoids (Figure 4c, inset). Based on their shape and location, these latter CNTF‐responsive cells resembled the resident macrophage population, also known as Kupffer's cells (Naito et al., 2004). CNTF response in Kupffer's cells was indeed confirmed by the presence of p‐STAT3 immunoreactive nuclei in some cells positive for the macrophage marker F4/80 (Lumeng et al., 2007) (Figure 4d). In conclusion, our data demonstrate that different cytotypes within the liver are responsive to systemic CNTF, given the profound induction of STAT3 phosphorylation in both the hepatocyte population and the sinusoidal immune–vascular component.

#### 3.2.4 Adipose tissues

**FIGURE 5:**
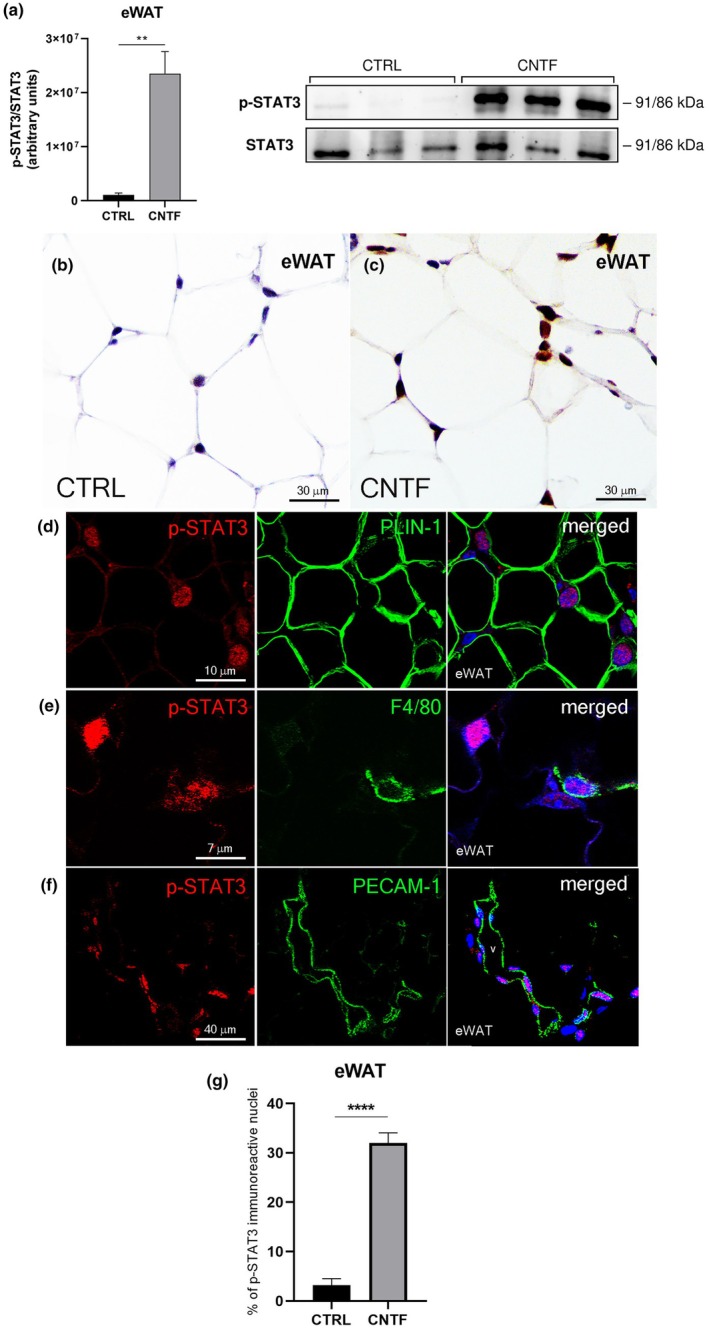
p‐STAT3 immunoreactivity after CNTF administration in murine white adipose tissue. (a) Representative Western blot and relative densitometric quantification of p‐STAT3 protein levels in the epididymal white adipose tissue (eWAT) of saline‐ (CTRL) and CNTF‐treated mice. Total STAT3 was used as a loading control. (b, c) Peroxidase immunohistochemistry for p‐STAT3 in the epididymal white adipose tissue after saline or CNTF administration. Double‐labelled confocal microscopy for p‐STAT3 and the lipid droplet‐associated protein (PLIN‐1) (d) or the macrophage marker F4/80 (e) or the endothelial marker Platelet/Endothelial Cell Adhesion Molecule‐1 (PECAM‐1) (f) in the epididymal white adipose tissue after CNTF administration. (g) Morphometric quantification of p‐STAT3‐positive nuclei in the eWAT. Data are mean ± SEM (*n* = 3 per group). **p < 0.01; ****p < 0.0001; Unpaired *t*‐test.

The epididymal fat depot surrounding the rodent male reproductive organs was collected as representative of the visceral WAT. Visceral WAT is the main site of lipid storage in mammals, and its expansion under conditions of increased caloric intake and/or reduced energy expenditure is strongly linked to the pathophysiology of obesity (Lumeng et al., 2007). Visceral WAT expansion progressively promotes liver metabolic dysfunction, systemic inflammation, and insulin resistance, thereby significantly contributing to morbid obesity and multi‐organ associated diseases (Lumeng et al., 2007). To evaluate the effect of the CNTF on this depot, we first analyzed eWAT total protein extracts via Western blotting. In saline‐treated mice, p‐STAT3 was nearly undetectable, whereas CNTF administration induced a significant increase in protein phosphorylation (Figure 5a). This activation was further detailed through immunohistochemical analysis: After saline treatment, p‐STAT3‐positive nuclei were not detected (Figure 5b), while CNTF administration resulted in a strong p‐STAT3 activation in eWAT (Figure 5c). Accordingly, morphometric analysis revealed a nearly absent activation of p‐STAT3 in saline‐treated mice (Figure 5g and Table 1), different from the widespread activation in CNTF‐treated mice, where the percentage of p‐STAT3‐reactive nuclei rose significantly to 32% ± 2% (vs 3% ± 1% in the CTRL, *p* < 0.0001; Figure 5g and Table 1). Most of the CNTF‐responsive cells were white adipocytes, as demonstrated by the presence of p‐STAT3 immunoreactive nuclei at the periphery of large unilocular cells that were also positive for the lipid droplet‐associated protein perilipin‐1 (PLIN‐1) (Figure 5d). p‐STAT3 immunoreactivity was observed also in the majority of cells positive for the macrophage marker F4/80 (Figure 5e) and in endothelial cells lining the lumen of numerous small blood vessels (arterioles, venules, and capillaries) and positive for the Platelet/Endothelial Cell Adhesion Molecule‐1 (PECAM‐1), an antigen expressed at the surface of endothelial cells (Lertkiatmongkol et al., 2016) (Figure 5f).

**FIGURE 6:**
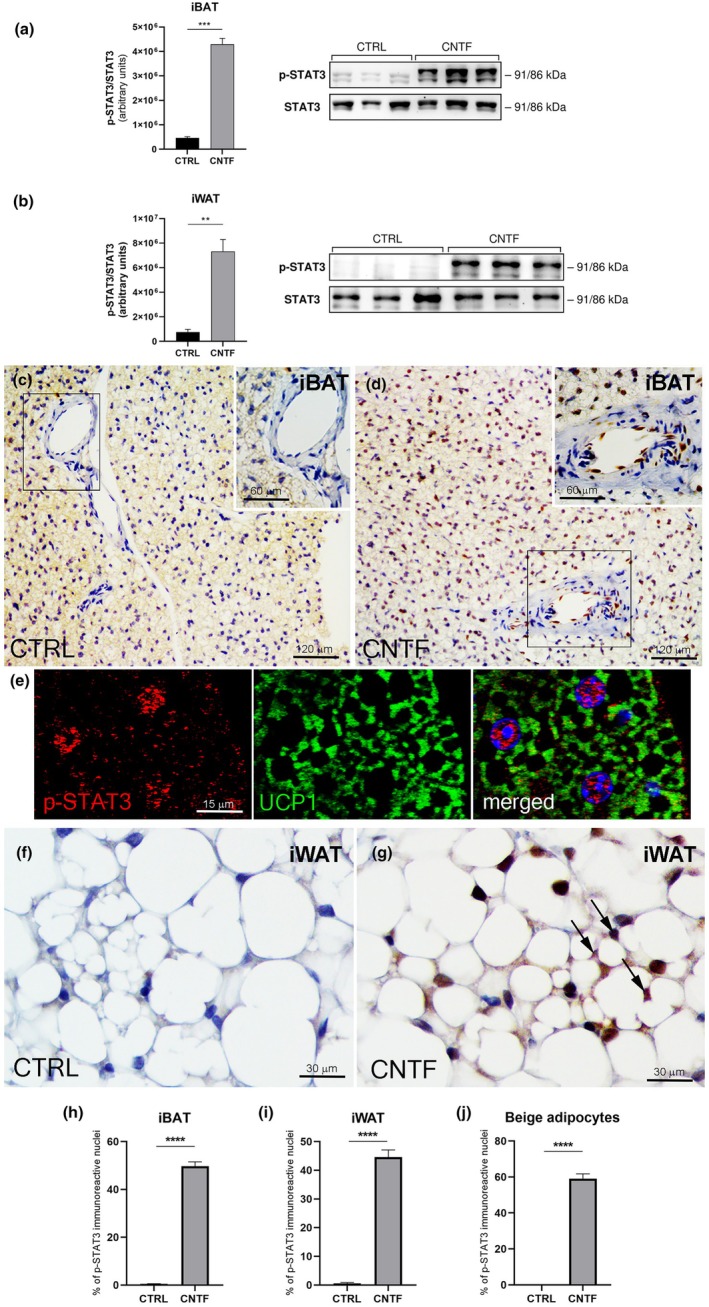
p‐STAT3 immunoreactivity after CNTF administration in murine brown and subcutaneous adipose tissue. (a) Western blot and densitometric analysis of p‐STAT3 levels in interscapular brown adipose tissue (iBAT) and (b) inguinal white adipose tissue (iWAT) of saline‐ (CTRL) and CNTF‐treated mice. Total STAT3 was used as a loading control. (c, d) Peroxidase immunohistochemistry for p‐STAT3 in the brown adipose tissue after saline or CNTF administration. Insets are enlargements of the corresponding framed area in (c) and in (d), with panel (d) showing p‐STAT3 immunoreactivity in small blood vessels, particularly arterioles, supplying the parenchyma. (e) Double‐labelled confocal microscopy for p‐STAT3 and the uncoupling protein 1 (UCP1) after CNTF administration. (f, g) Peroxidase immunohistochemistry for p‐STAT3 in the subcutaneous white adipose tissue after saline or CNTF administration. Figure (g) shows a CNTF‐dependent STAT3 phosphorylation in paucilocular beige adipocytes (black arrows). (h) Morphometric quantification of the percentage of p‐STAT3‐positive nuclei in iBAT, iWAT, and beige adipocytes. Data are expressed as mean ± SEM (*n* = 3 per group). **p < 0.01; ***p < 0.001, ****p < 0.0001; Unpaired *t*‐test.

In small rodents, the interscapular adipose tissue is mainly composed of brown adipocytes, whose thermogenic activity is required to perform thermoregulation, but also affects energy expenditure and body weight (Frontini & Cinti, 2010). Western blot analysis in the iBAT revealed an increased STAT3 phosphorylation following systemic CNTF administration (Figure 6a). In the iBAT of saline‐injected mice, a weak expression of p‐STAT3 was detected in the cytoplasm and in very few cellular nuclei by immunohistochemistry (Figure 6c). A strong STAT3 phosphorylation was observed after CNTF administration in numerous multilocular adipocytes (Figure 6d). Such a response was confirmed by morphometric analysis, revealing a negligible basal activation in saline‐treated mice (Figure 6h and Table 1) compared to a massive response in CNTF‐treated mice with 49% ± 2% of nuclei becoming p‐STAT3‐positive (vs 0.4% ± 0% in the CTRL, *p* < 0.0001; Figure 5h and Table 1). In treated mice, P‐STAT3‐specific staining was mainly detected in uncoupling protein 1 (UCP1)‐positive brown adipocytes (Figure 6e). As already observed for the eWAT, however, CNTF administration also led to p‐STAT3 immunoreactivity in different cells belonging to the wall of numerous small blood vessels supplying the parenchyma (Figure 6d, inset).

In rodents, the subcutaneous iWAT is the largest and most relevant fat depot capable of recruiting beige adipocytes, that is, brown‐like adipocytes, that derive both from de novo adipogenesis and/or from the transdifferentiation of white into brown‐like cells under proper stimuli (Barbatelli et al., 2010; Wang et al., 2013). Similarly to the iBAT, a strong induction of STAT3 phosphorylation was detected in CNTF‐treated mice by Western blotting (Figure 6b). Consistently, whereas p‐STAT3 immunoreactivity was virtually absent in saline‐injected mice (Figure 6f), CNTF treatment led to STAT3 phosphorylation in numerous subcutaneous white and beige adipocytes, these latter identified by their distinctive paucilocular morphology (Barbatelli et al., 2010) (Figure 6g). Morphometric analysis confirmed that p‐STAT3‐immunoreactive nuclei were nearly absent in the controls (Figure 6i,j and Table 1), whereas CNTF treatment resulted in a significant increase reaching 44% ± 2% (vs 1% ± 0% in the CTRL, *p* < 0.0001) and 59% ± 2% (vs 0% ± 0% in the CTRL, *p* < 0.0001) of white and beige adipocytes, respectively (Figure 6i,j and Table 1). As observed for eWAT, p‐STAT3 immunoreactivity was detected also in several small blood vessels supplying the tissue, but never in macrophages.

In conclusion, CNTF administration massively induced STAT3 phosphorylation in visceral white adipocytes of eWAT, in brown adipocytes of iBAT, and in white and beige adipocytes of iWAT. In these tissues, CNTF also targeted small blood vessels supplying the adipose depots and, only in the visceral fat, the resident macrophage population.

#### 3.2.5 Skeletal muscle

**FIGURE 7:**
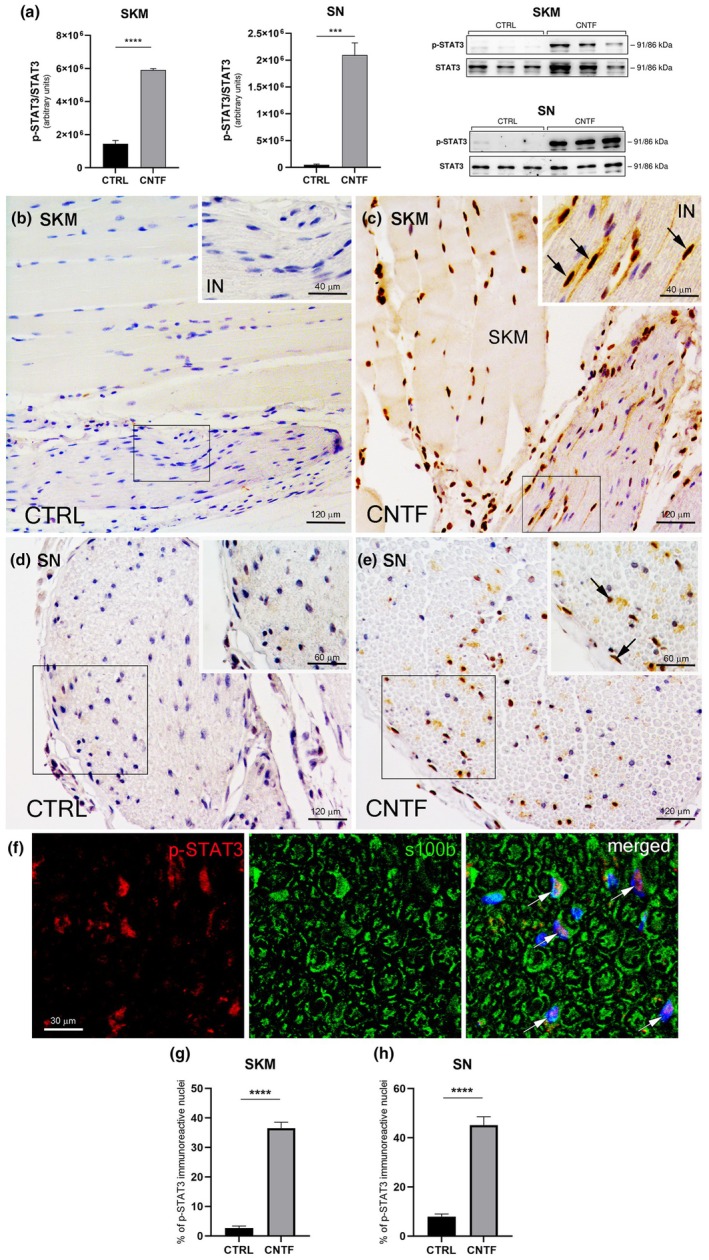
p‐STAT3 immunoreactivity after CNTF administration in murine skeletal muscle. (a) Representative Western blot and densitometric quantification of p‐STAT3 protein levels in the gastrocnemius skeletal muscle (SKM) and sciatic nerve (SN) of saline‐ (CTRL) and CNTF‐treated mice. Total STAT3 was used as a loading control. (b, c) Peroxidase immunohistochemistry for p‐STAT3 in the gastrocnemius after saline or CNTF administration. Insets are enlargements of the corresponding framed area in (b, c) and in figure (c) shows p‐STAT3 immunoreactivity in peripheral motor nerves innervating the gastrocnemius (black arrows). (d, e) Peroxidase immunohistochemistry for p‐STAT3 in the sciatic nerve after saline or CNTF administration. Insets are enlargements of the corresponding framed area in (d, e) and in figure (e) shows p‐STAT3 immunoreactivity in nerve cells and in cells of the epineurium (black arrows). (f) Double‐labelled confocal microscopy for p‐STAT3 and the Schwann's cells marker S100b in sciatic nerve after CNTF administration, showing S100b‐positive cells immunoreactive for p‐STAT3 (white arrows). (g) Morphometric quantification of the percentage of p‐STAT3‐immunoreactive nuclei in skeletal muscle and sciatic nerve. Data are expressed as mean ± SEM (*n* = 3 per group). ***p < 0.001, ****p < 0.0001 (Unpaired *t*‐test). IN, Intramuscular peripheral nerve.

To assess the effect of CNTF on the skeletal muscle, we evaluated the activation of the JAK/STAT3 pathway in the gastrocnemius muscle. By Western blotting, systemic CNTF administration induced a significant increase in p‐STAT3 protein levels compared to saline (Figure 7a). In the gastrocnemius muscle of the saline‐treated mice, p‐STAT3 immunoreactivity was virtually absent, with only a few scattered elongated nuclei showing weak basal phosphorylation (Figure 7b). In contrast, CNTF administration triggered a robust and widespread response (Figure 7c). Specifically, strong nuclear p‐STAT3 immunoreactivity was observed in 36% ± 2% (vs 3% ± 1% in the CTRL, *p* < 0.0001; Figure 7g and Table 1) of the peripheral nuclei of striated muscle fibers, which are characteristic of these multinucleated syncytial structures. As observed in the adipose tissue, CNTF also induced STAT3 phosphorylation in the endothelial cells of blood vessels supplying the skeletal muscle. A particularly striking response was observed in the intramuscular nerves supplying the gastrocnemius, where numerous p‐STAT3‐positive nuclei were detected in CNTF‐treated animals (Figure 7c, inset).

To further characterize the effect of CNTF on peripheral nerves, we extended our analysis to the sciatic nerve, the prototypical peripheral nerve, containing both sensory and motor axons. Western blot analysis revealed a significant increase of p‐STAT3 protein expression in the sciatic nerve of CNTF‐treated mice compared to saline (Figure 7a). Immunohistochemical analysis (Figure 7d,e) and subsequent morphometric quantification revealed a massive induction of nuclear p‐STAT3 in the treated group, reaching 45% ± 3% (vs 8% ± 1% in the CTRL, *p* < 0.0001; Figure 7h and Table 1). CNTF was found to induce p‐STAT3 phosphorylation in the epineurium cells (a layer of connective tissue surrounding nerve fascicles) and in cells colocalizing with S100b (Figure 7f), a marker for Schwann's cells, the glial cells involved in axon's support, survival, and myelination. Most CNTF‐activated cells located in the intramuscular nerves were S100b‐positive Schwann cells.

In conclusion, our data demonstrate that systemic CNTF exerts a powerful effect on skeletal muscle, targeting both the contractile fibers and its vascular component. Importantly, CNTF also acts on the skeletal muscle nerve supply, lending support to a possible wide action on peripheral nerves.

## 4 DISCUSSION

CNTF is a neurotrophic factor promoting neuronal survival and differentiation (Arakawa et al., 1990; Oyesiku & Wigston, 1996). Our group has previously demonstrated that exogenous CNTF effectively targets the CNS, bypassing the blood–brain barrier at specific circumventricular organs, such as the median eminence and the area postrema, where it mainly triggers the activation of the JAK2‐STAT3 signaling pathway (Senzacqua et al., 2016; Severi et al., 2015; Venema et al., 2020). Furthermore, CNTF was demonstrated to promote leptin entry into hypothalamic feeding centers and to display anorexigenic activity (Lambert et al., 2001; Sleeman et al., 2003; Venema et al., 2020). At the same time, recent evidence has suggested broader actions for this cytokine outside the CNS, targeting metabolically relevant peripheral organs (Pasquin et al., 2015). It should be noted, however, that much of the current knowledge regarding peripheral CNTF activity stems from in vitro studies.

In this study, by combining morphological and quantitative approaches, we provided the first comprehensive in vivo map of CNTF‐responsive cells, using STAT3 phosphorylation as a functional proxy due to the limited availability of reliable antibodies against the Cntfrα (Severi et al., 2015; Venema et al., 2020; Zvonic et al., 2003). Indeed, both central and peripheral effects of exogenous CNTF are significantly blunted or abolished when STAT3 signaling is pharmacologically or genetically inhibited, while remaining largely unaffected by the blockade of alternative CNTF‐induced pathways such as ERK1/2‐MAPK or AKT (Rezende et al., 2009).

Our in vivo mapping revealed a widespread activation of STAT3 signaling across the GI tract. The discrepancy we observed between robust p‐STAT3 signaling and negligible *Cntfrα* transcripts in the alimentary system suggests a high signaling efficiency at low receptor densities or, alternatively, the involvement of alternative signaling modes, such as the “trans‐signaling” (Rose‐John, 2018). This mechanism, shared with other IL‐6 family members, allows the circulating complex formed by CNTF and its soluble receptor to activate cells expressing only gp130 and Lifrβ, thereby extending the cytokine's action even to tissues with low expression of the membrane‐bound Cntfrα. Our mapping identified the smooth muscle cells of the muscularis externa as the primary site of STAT3 activation within the GI tract. While previous studies focused on STAT3 in the mucosal epithelium as a mediator of inflammation (Bollrath et al., 2009; Pickert et al., 2009), its activation within the other layers remains largely unexplored in vivo. Beyond the muscularis layer, we documented significant p‐STAT3 activation in the lamina propria and in the submucosal and myenteric plexuses. These findings align with reports that CNTF promotes the survival of myenteric neurons in vitro (Kato et al., 2024). The coordinated activation of STAT3 in the enteric nervous system and contractile layers suggests a direct modulation of the GI motility and provides a novel possible explanation for the nausea and vomiting observed in clinical trials, which were previously attributed solely to central actions in the area postrema (Ettinger et al., 2003; Senzacqua et al., 2016). Our study further extended to the major extramural glands associated with the GI tract, specifically the pancreas and the liver. Regarding the pancreas, our quantitative analysis revealed a prominent p‐STAT3 activation in the endocrine pancreas. Our morphological data agree with the present literature reporting that the STAT3 signaling is a well‐established transducer of CNTF action in pancreatic islets in vitro (Rezende et al., 2007, 2009). Notably, we showed for the first‐time activation of glucagon‐producing α‐cells alongside insulin‐producing β‐cells, suggesting coordinated effects on glucose homeostasis involving both cell types. Similarly, in the liver we observed a significant p‐STAT3 induction within hepatocytes, Kupffer cells and sinusoids. Since hepatic STAT3 activation is a key regulator of glucose and lipid metabolism—specifically by suppressing gluconeogenic gene expression and enhancing insulin sensitivity (Inoue et al., 2004)—our data added an important interpretative insight into the well‐established metabolic effects of the CNTF in the liver (Cui et al., 2017; Sleeman et al., 2003). A notable finding of our mapping is the recruitment of STAT3 signaling within several immune populations, including adipose tissue macrophages. As described by Akira (2000), STAT3 plays a key role in immune cells, often mediating anti‐inflammatory and pro‐survival roles. In the context of adipose tissue, STAT3 activation occurred in both macrophages and adipocytes, where it has been linked to lipolysis and browning (Cernkovich et al., 2008; Derecka et al., 2012), suggesting that the well‐described anti‐obesity effects of CNTF in vitro (Perugini et al., 2019; Zvonic et al., 2003) may involve a direct modulation of the adipose tissue microenvironment and its inflammatory state in vivo. For the first time, we documented a significant JAK2‐STAT3 activation in beige adipocytes, further suggesting that CNTF may involve the recruitment of thermogenic programs across different adipose depots. Lastly, as a major site of energy substrate utilization, we showed a significant STAT3 phosphorylation in the skeletal muscle, where CNTF is known to improve insulin sensitivity, increase protein synthesis (Wang & Forsberg, 2000), and enhance fatty acid utilization (Watt et al., 2006; Zvonic et al., 2003). Our study showed that, within skeletal muscle, STAT3 signaling is also activated in Schwann cells of peripheral nerves supplying the tissue, including the sciatic nerve. Such response in glial cells—a major source of endogenous CNTF—suggests a potential autocrine/paracrine loop (Lee et al., 1995).

The biological outcome of STAT3 signaling is critically dependent on the dosage and its temporal dynamics. In our model, the acute recruitment of STAT3 signaling reflects a physiological stimulus generally considered homeostatic and protective, differing significantly from the persistent overactivation associated with chronic inflammatory diseases and metabolic dysfunction (Haikarainen et al., 2026; Rose‐John, 2018).

While our study defines an anatomical fingerprint of acute CNTF administration, we acknowledge some limitations. First, as our study is observational in nature, the functional implications of these findings are inferred based on existing literature. Second, our analysis was restricted to male subjects, thus not accounting for sex‐specific responses previously documented (Colleluori et al., 2025; Perugini et al., 2022). Lastly, an additional limitation is that CNTF can induce IL‐6 release, which in turn activates the same signaling cascade. Although this process occurs later than the 45 min window used in our protocol, we cannot entirely exclude the influence of the endogenous cytokine background on STAT3 phosphorylation (Hu et al., 2020). In conclusion, this anatomical mapping underscores the necessity of considering cell‐type‐specific responses to understand the therapeutic potential and side‐effect profile of CNTF‐based treatments. By identifying novel peripheral targets, we provide a morphological framework for future functional studies on cytokine‐mediated metabolic regulation.

## AUTHOR CONTRIBUTIONS

**Chiara Galli:** concept/design, acquisition of data, data analysis/interpretation, drafting of the manuscript; **Georgia Colleluori:** acquisition of data, data analysis/interpretation, drafting of the manuscript; **Jessica Perugini, Edoardo Scopini, Ilenia Severi, and Gaia Grandin:** acquisition of data, data analysis/interpretation; **Antonio Giordano:** concept/design, critical revision of the manuscript, and approval of the article.

## CONFLICT OF INTEREST STATEMENT

The authors declare that they have no competing interests.

## Supporting information


**Figure S1:** p‐STAT3 immunoreactivity after CNTF administration in murine colon. (a) Western blot and densitometric analysis of p‐STAT3 protein levels in the colon of saline‐ (CTRL) and CNTF‐treated mice. Total STAT3 was used as a loading control. (b, c) Immunohistochemical staining showing the distribution of p‐STAT3‐positive nuclei in the colon. (d) Morphometric analysis of p‐STAT3‐positive nuclei across the lamina propria (LP) submucosa (SM), and muscularis layer (ML) of the colon. Data are mean ± SEM (*n* = 3 animals per group). ***p < 0.001, ****p < 0.0001 (unpaired *t*‐test).

**Figure S2:** CNTF response in incretin‐producing enteroendocrine cells in murine intestine. Double‐labelled confocal microscopy for p‐STAT3 and the glucagon‐like peptide 1 (GLP‐1) (a), or the gastric inhibitory peptide (GIP) (b) in the mesenteric gut after CNTF administration. M = mucosa, Mus = muscularis externa, LP = lamina propria.

**Table S1:** Taqman probes.
**Table S2a:** Primary antibodies.
**Table S2b:** Secondary antibodies.

## Data Availability

The data that support the findings of this study are available from the corresponding author upon reasonable request.
