# Supplementary figures and images for: The ciliary neurotrophic factor induces Stat3 phosphorylation in distinctive cytotypes of organs involved in body metabolism: An immunohistochemical study
Source: J Anat. 2026 Jun 25:10.1111/joa.70195. Online ahead of print. doi: 10.1111/joa.70195 (PMC13398739; doi:10.1111/joa.70195)

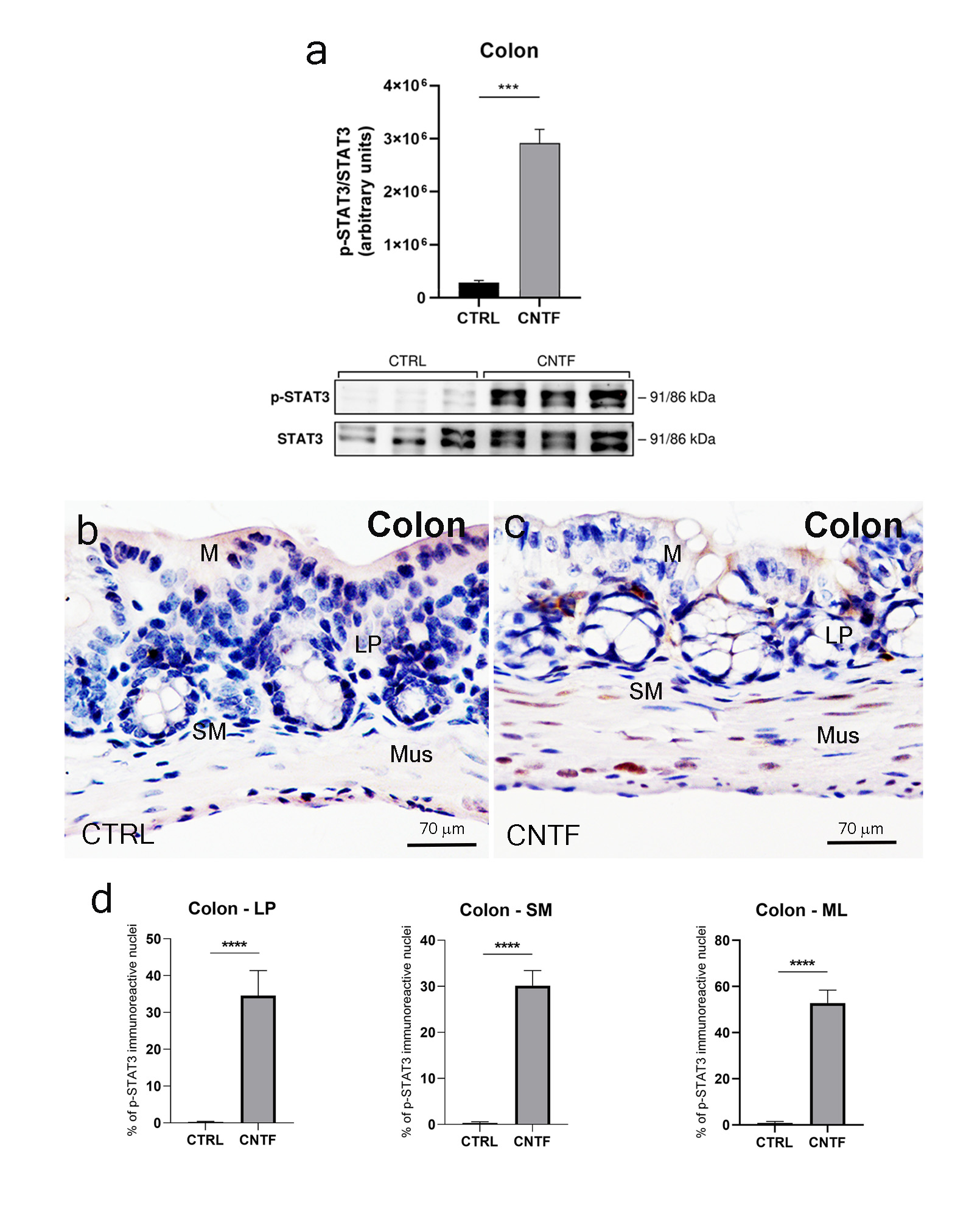

Supplement: Supplementary file 1 — Figure S1: p‐STAT3 immunoreactivity after CNTF administration in murine colon. (a) Western blot and densitometric analysis of p‐STAT3 protein levels in the colon of saline‐ (CTRL) and CNTF‐treated mice. Total STAT3 was used as a loading control. (b, c) Immunohistochemical staining showing the distribution of p‐STAT3‐positive nuclei in the colon. (d) Morphometric analysis of p‐STAT3‐positive nuclei across the lamina propria (LP) submucosa (SM), and muscularis layer (ML) of the colon. Data are mean ± SEM (n = 3 animals per group). ***p < 0.001, ****p < 0.0001 (unpaired t‐test). [file JOA-9999-0-s003.zip › Fig. S1 - REVISED_2.jpg]

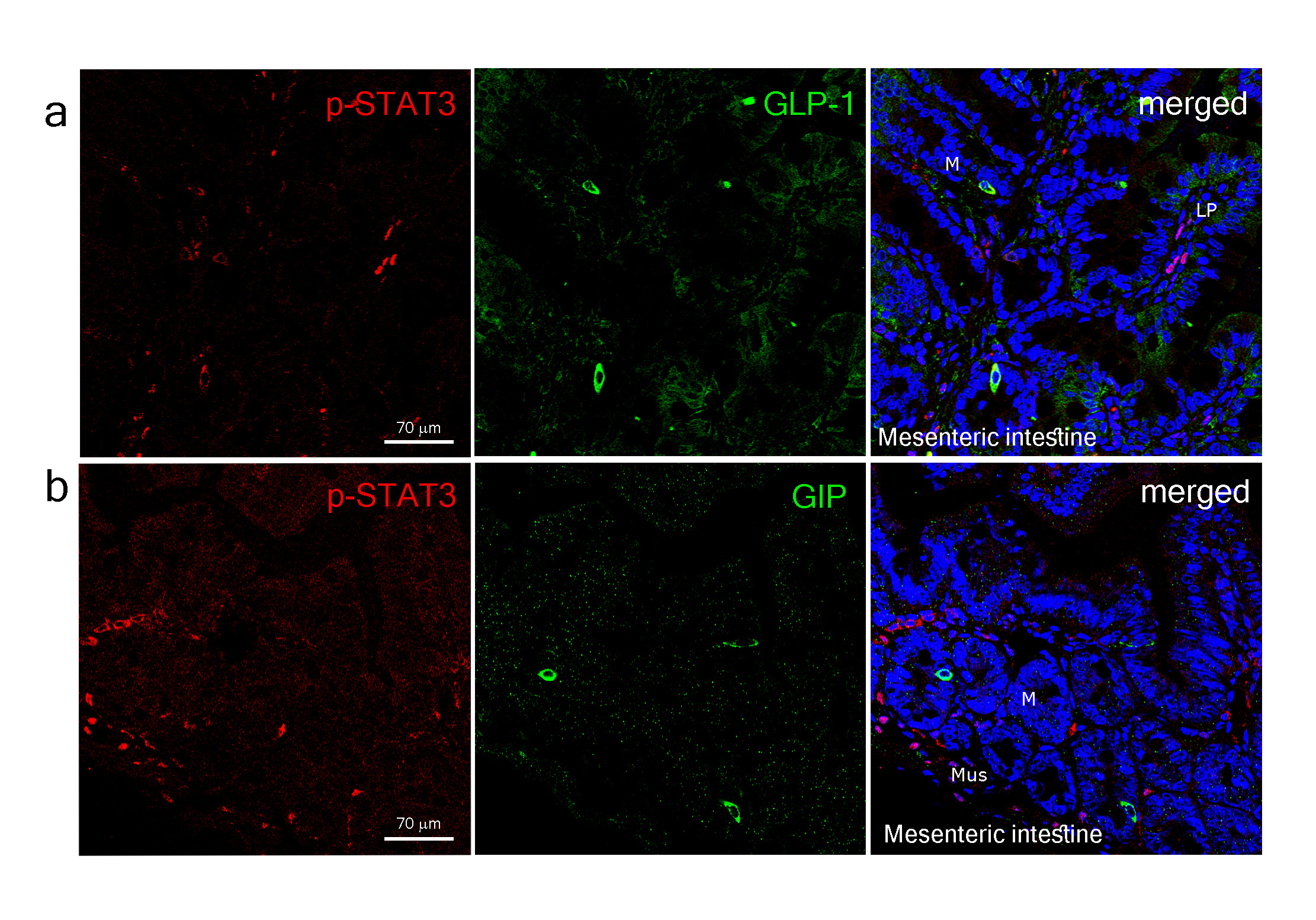

Supplement: Supplementary file 2 — Figure S2: CNTF response in incretin‐producing enteroendocrine cells in murine intestine. Double‐labelled confocal microscopy for p‐STAT3 and the glucagon‐like peptide 1 (GLP‐1) (a), or the gastric inhibitory peptide (GIP) (b) in the mesenteric gut after CNTF administration. M = mucosa, Mus = muscularis externa, LP = lamina propria. [file JOA-9999-0-s002.zip › Fig. S2 - REVISED_2.jpg]
